# Five years of ChemRxiv: where we are and where we go from here†

**DOI:** 10.1039/d2sc90224a

**Published:** 2022-12-02

## Abstract

ChemRxiv was launched on August 15, 2017 to provide researchers in chemistry and related fields a home for the immediate sharing of their latest research. In the past five years, ChemRxiv has grown into the premier preprint server for the chemical sciences, with a global audience and a wide array of scholarly content that helps advance science more rapidly. On the service’s fifth anniversary, we would like to reflect on the past five years and take a look at what is next for ChemRxiv.

## Abstract 2

On the service’s fifth anniversary, we would like to reflect on the past five years and take a look at what is next for ChemRxiv.

## Graphical abstract

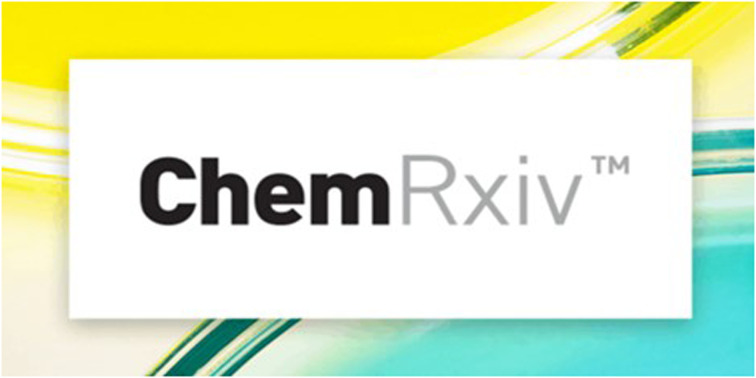

Because of the importance of serving researchers around the world, ChemRxiv operates as a collaboration among five of the largest chemical societies: the American Chemical Society, the Chemical Society of Japan, the Chinese Chemical Society, the German Chemical Society (Gesellschaft Deutscher Chemiker), and the Royal Society of Chemistry. ChemRxiv is also guided by a 29-member Scientific Advisory Board composed of researchers from 17 countries.

With the participation of these governing organizations and advocates, ChemRxiv helps unite the chemistry community and open up its scholarship. The shared goals for ChemRxiv remain the same as when it was launched:

• Rapid sharing of initial research results, accelerating the time for key information to reach the broader community and leading to new collaborations

• Appropriate credit for authors’ work

• Extended reach for the latest research findings

• Visibility into completed research for job applications, tenure packages, grant proposals, *etc.*

• Opportunity for community feedback on an early manuscript version, leading to a stronger peer-reviewed article

We recently surveyed over 900 chemists to learn more about their views on preprints, and over 43% rated “**staking the first claim to new research**” as an extremely important benefit, with more than 87% rating it at least somewhat important. This places the desire to get proper credit at the top of the list of benefits, followed by “**rapid sharing of results to the community**” and then “**public record of research activity**”. We will continue to engage with our authors in the years ahead to ensure that our goals align with the benefits that they value most.

## The past five years

**Fig. 1:**
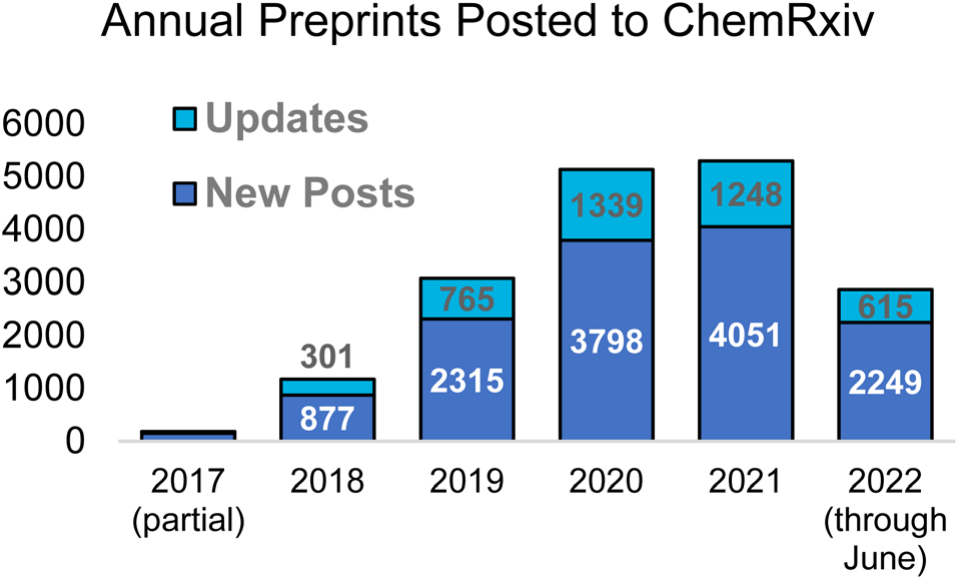
Total preprints posted on ChemRxiv per year since launch. Data for 2022 are through June 30.

**Fig. 2:**
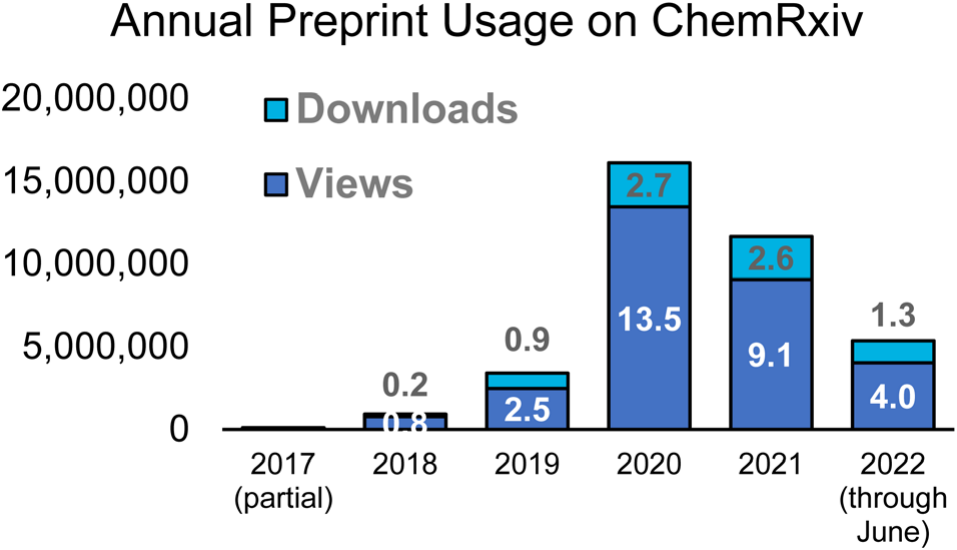
Total views and downloads of ChemRxiv preprints per year since launch. Data label values are in millions. Data for 2022 are through June 30.

The posting of preprints in chemistry has steadily grown over the past five years – see Fig. 1 for annual numbers of posted preprints on ChemRxiv. There were also noticeable boosts in the reading of preprints during the early phases of the COVID pandemic, when many labs were shut down and researchers in all fields were interested in uncovering new data that could later lead to important treatments and vaccines (see Fig. 2). Consumption of ChemRxiv content has slowed slightly after reaching a high, resulting from some highly-read COVID preprints, and the number of preprints posted in 2022 is on pace for almost a 10% increase over 2021.

When considering how the posted preprints fit into the larger publishing ecosystem, we have found that more than half of the nearly 14 000 preprints on ChemRxiv have already been published in peer-reviewed journals, and this number continues to grow. The final publication destinations for ChemRxiv authors include the full range of top journals in chemistry, such as titles published by all our partner societies, and even multidisciplinary journals like *Nature* and *Science*.

In the same survey described above, the top concern about preprints was that they could be used to spread misinformation (over 65% of 922 respondents listed this as at least somewhat concerning). This result leads us to continue to emphasize our screening process and our outward messaging. While intentionally not approaching the rigor of peer review to expedite the posting process, every preprint submitted to ChemRxiv is reviewed by a PhD-level chemist for basic appropriateness and lack of any harmful or overtly fraudulent content. We have also carefully labeled the preprint page and our homepage with a warning that the content has not yet been peer reviewed and should not be considered final. As we increasingly engage with the preprint community and general public, we will take care to balance the rapid sharing of new content with the concern over incorrect information being spread.

## ChemRxiv today

ChemRxiv readers and authors are now just over one year into using a new and improved platform hosted on Cambridge Open Engage. Designed specifically for preprints, the new site offers excellent tools for screening incoming submissions, improved webpages for posted preprints, and a continuously evolving API that connects ChemRxiv with many other downstream indexing services. These tools and features combine to help us rapidly post new preprints (often in one business day) and then ensure that those preprints are found by a wide audience.

We have also continued to expand ChemRxiv’s Direct Journal Transfer service, which allows authors to transfer their preprint manuscript directly to a peer-reviewed journal. Launched with journals from three publishers in 2018, the service now includes 152 possible destination journals from six publishers, with more on the way.

Based on community feedback, ChemRxiv also now accepts reviews, moving beyond its initial scope of full-length research manuscripts alone. This expansion of ChemRxiv’s remit has proven popular, with over 130 reviews posted in the first three months, providing even more opportunities for chemists to have an outlet to share their scholarship for feedback and credit.

## The future of ChemRxiv

Now at ChemRxiv’s fifth anniversary, it is important to consider how preprints will fit into the publishing process for years to come. All the benefits of preprints described above are valid today, and we will continue to invest in improvements to ChemRxiv. Based heavily on input from our community, our near-term efforts will focus on several areas:

(1) Improving the experience for authors and readers using the ChemRxiv site, both to submit new preprints and discover new posted content

(2) Connecting more closely to the broader publishing space, for example by better sharing metadata to link preprints to published journal articles and ORCID profiles

(3) Continuing to improve the infrastructure underlying the site so we can keep processing times low and make it easy to find relevant preprints

While we are encouraged by the steadily growing number of preprints posted on ChemRxiv, we see plenty of room to broaden the adoption of preprints across chemistry. We are continuing to expand the geographic representation on our Scientific Advisory Board and to engage with researchers from diverse fields, career stages, and locations to better support the full breadth of the chemistry community.

## Thank you!

All of us on the ChemRxiv team are grateful for the many authors and readers who have supported the service over these past five years. As preprints become more commonplace in the chemical sciences, we hope to remain a top destination for cutting-edge research findings. If you have ideas for how ChemRxiv can better serve the community, we would love to hear from you. Feedback is always welcome *via* email at curator@chemrxiv.org.

As we move into the next five years and beyond, we hope that you will join the movement by routinely posting preprints of your work (if you don’t already!) and sharing any interesting preprints that you read. The five societies that manage ChemRxiv are united in their belief that the rapid interchange of scientific results benefits not only chemists but the broader world. Preprints are a valuable step on the path to publication, and we encourage you to take advantage of the many benefits to sharing your work in this way. Our shared belief in the value of preprints is why ChemRxiv was launched, why it is still here today, and why we’ll continue to adapt it to the needs of our community in the years ahead.

 

**Benjamin Mudrak, PhD**, Senior Product Manager, ChemRxiv, ORCiD: 0000-0002-2805-5690

**Sara Bosshart**, Head of Open Access Journals, Royal Society of Chemistry

**Prof. Dr Wolfram Koch**, Executive Director, German Chemical Society

**Allison Leung**, Senior Manager, Product Development, ACS Publications

**Donna Minton, PhD**, Director of Publications, Chinese Chemical Society

**Mitsuo Sawamoto, PhD**, Executive Director, Chemical Society of Japan

**Sarah Tegen, PhD**, Senior Vice President Journals Publishing Group, ACS Publications

## Corresponding Author

Benjamin Mudrak, PhD

Senior Product Manager, ChemRxiv

curator@chemrxiv.org

## Notes

The authors are employed by the organizations that co-own and manage ChemRxiv.

## Supplementary Material

